# MALT Lymphoma of the Bladder: A Case Report and Review of the Literature

**DOI:** 10.1155/2015/934374

**Published:** 2015-08-31

**Authors:** Prashant Vempati, Miriam A. Knoll, Mahfood Alqatari, James Strauchen, Adriana K. Malone, Richard L. Bakst

**Affiliations:** ^1^Icahn School of Medicine at Mount Sinai, New York, NY 10029, USA; ^2^Department of Radiation Oncology, Icahn School of Medicine at Mount Sinai, New York, NY 10029, USA; ^3^Department of Pathology, Icahn School of Medicine at Mount Sinai, New York, NY 10029, USA; ^4^Department of Medical Oncology, Icahn School of Medicine at Mount Sinai, New York, NY 10029, USA

## Abstract

The presentation of a MALT lymphoma in the bladder is exceedingly rare. Furthermore, the optimal treatment of primary MALT confined to the bladder remains to be defined. Here, we report a case of a 65-year-old female with primary MALT lymphoma treated with definitive radiation therapy. The patient received a total dose of 30 Gy in 20 equal daily fractions to the bladder and tolerated the treatment well. In addition, we have extensively reviewed the relevant literature to better define the optimal management of this rare disease. In conclusion, primary MALT lymphoma of the bladder represents a rare malignancy with excellent prognosis if detected at an early stage. For early stage disease, definitive radiation represents an excellent treatment modality with a minimal side-effect profile.

## 1. Introduction

The majority of bladder cancers are epithelial in origin [1, 2]. Lymphomas of the urinary bladder are exceedingly rare and can be divided into (i) primary, a rare lymphoma arising in the urinary bladder with no evidence of lymphoma elsewhere, or (ii) secondary lymphoma of the urinary bladder associated with lymphoma at an extra vesicle site [3, 4]. Primary malignant lymphoma of the bladder accounts for less than 1% of neoplasms unlike secondary lymphoma, which is much more common [1, 2]. Of primary lymphomas of the bladder, mucosa associated lymphoid tissue lymphoma, or MALT, is the most prevalent histological subtype. The optimal treatment of primary MALT confined to the bladder remains to be defined. Here, we report a patient treated with definitive radiation and review the relevant literature to better define the optimal management of this rare disease.

## 2. Case Report

**Figure 1:**
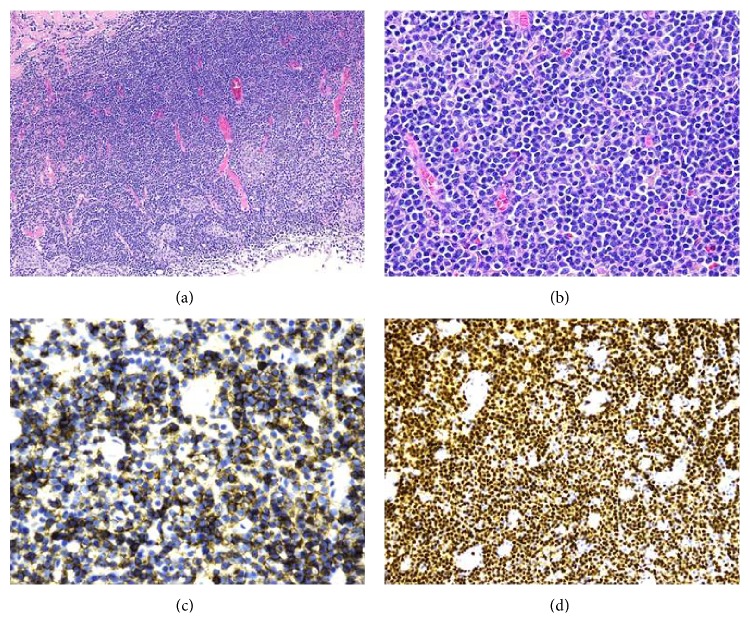
(a) Low power images of H&E stain show infiltration of the bladder wall by small plasmacytoid lymphocytes. (b) High power images of H&E stain show infiltration of the bladder wall by small plasmacytoid lymphocytes. (c) CD20 stain shows positive cells. (d) PAX-5 stain shows diffusely positive cells.

A 65-year-old female presented to her gynecologist after she noted a two-week history of spotting on toilet paper after urinating. She was referred to a urologist for further investigation of the bladder lesion. A transurethral resection of the bladder tumor (TURBT), with resection of the posterior bladder wall, right bladder wall, and bladder neck, was performed at an outside hospital. Initial pathology based on H&E stain and immunohistochemistry favored extra nodal marginal zone lymphoma with follicular colonization, with cells positive for CD20 and PAX-5 and negative for CD5 and CD10 (Figure 1). Laboratory evaluation including LDH, B2-microglobulin, serum immunofixation, and protein electrophoresis was all within normal limits. Subsequently, the patient underwent a PET/CT and bone marrow biopsy, and there was no evidence of any extra vesicular disease.

The patient was referred to radiation oncology to discuss the role of definitive radiation in her treatment regimen. Magnetic resonance imaging and repeat cystoscopy were recommended and performed to assess the presence of residual disease and were both negative. A well-healed biopsy area was noted on cystoscopy consistent with the site of original lesion. At this time, the patient's hematuria was resolved and she denied any weight loss, fatigue, night sweats, or fevers. The patient had no history of recurrent sexually transmitted diseases. There was no lymphadenopathy, organomegaly, or abnormal findings on physical examination.

**Figure 2:**
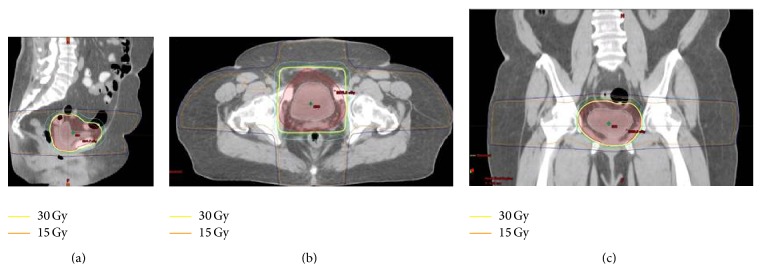
3D conformal radiation therapy of MALT lymphoma of the bladder with dose distributions depicted in the sagittal (a), axial (b), and coronal (c) planes.

Based on patient's lack of symptoms, negative imaging, and negative repeat cystoscopy, the patient was offered a course of close observation with serial cystoscopic evaluations versus definitive radiation given that the presence of microscopic disease could not be ruled out. She elected to proceed with radiation therapy. The patient received a total dose of 30 Gy in 20 equal daily fractions with a 4-field 3D-CRT plan utilizing PH16 MV photons. The adjacent normal structures were shielded with a multileaf collimator (MLC) (Figure 2). The patient tolerated radiation treatment well. She had no gastrointestinal, urinary, or gynecological toxicities during treatment and at short interval follow-up. Repeat evaluation 3 months following radiation with PET/CT revealed no evidence of disease and urine cytology was also negative.

## 3. Discussion

Primary lymphoma of the bladder is a rare malignancy, with limited literature to guide therapy. The first ever reported case of bladder lymphoma was reported in the literature by Eve and Chaffey in 1885 [4–8]. There have been less than 100 cases described in the literature since [4, 9, 10]. The disease typically presents in the 6th decade of life with slight predominance in females [6, 10]. Since lymphoid tissue is not found in the urinary bladder, chronic inflammation is postulated as the origin. As such, most patients present with symptoms of chronic cystitis [9]. However, similar to our patient, there have been many reported cases in which chronic cystitis and histological evidence of inflammation are lacking [4, 6, 11, 12]. The most common symptoms of lymphoma of the urinary bladder include weight loss, fatigue, hematuria, dysuria, nocturia, urinary frequency, and suprapubic or abdominal pain [4, 11, 12].  Table 1 contains a summary of basic patient demographics, presenting symptoms, treatments, and outcomes of all reported cases of primary MALT lymphoma of the bladder.

There are many treatment options for nongastric MALT lymphomas: (i) observation (based on factors such as patient age, risk factors, and tumor grade, observation might be the best option [11]); (ii) surgery (complete excision or biopsy) (MALT lymphomas that are unifocal can be partially or completely removed with procedures like TURBT [11]); (iii) radiation (radiation, when given alone or after an excisional biopsy, has shown excellent local control and improved overall disease-free survival [10, 13–15]); (iv) chemotherapy (usually used when a patient presents with systemic involvement or secondary lymphoma of the bladder [16–18]); (v) targeted antibody therapy (anti-CD20 antibody (rituximab) has been used along with other modalities in systemic lymphomas [17, 19]); (vi) antibiotics (they are usually used in cases where there is a known bacterial origin such as* H. pylori* in the stomach). There have been rare cases where antibiotics were used for MALT lymphomas of the bladder [20].

The presentation of bladder MALT lymphoma is exceedingly rare; however, MALT lymphomas at other sites are common, especially in the GI tract, salivary gland, lung, Waldeyer's ring, ocular adnexa, skin, thyroid, and breast. These lymphomas are highly radiosensitive. For localized disease, radiotherapy is the most appropriate treatment for organ preservation. It should be noted that, in patients of reproductive age, there is a risk of infertility with definitive radiation to the bladder secondary to the proximity of nearby reproductive organs. In these cases, maximal resection with TURBT may be the best treatment option when fertility is of concern. Chemotherapy and rituximab are reserved for secondary, recurrent, or disseminated disease [16–18, 21]. Al-Maghrabi et al. in 2001 identified four patients who received low dose radiotherapy for Stage IAE primary lymphoma of the bladder in a 30-year retrospective study. All four patients are alive and recurrence-free 2–13 years after treatment [10].

When considering radiation as a definitive monotherapy, staging becomes of utmost importance. 18F-FDG PET/CT, pelvic MRI, and bone marrow biopsy are used for initial disease staging and to rule out disseminated disease [1, 2, 10, 12]. As there was no evidence of disease in our patient's imaging work-up and post-TURBT cystoscopy, she was presented with observation with cystoscopy at 3-4-month intervals versus radiation therapy with less frequent cystoscopy and imaging. The optimal follow-up strategy for patients with lymphoma of the bladder remains unknown. In our practice, once the patient achieves complete response on posttreatment imaging, no further imaging is indicated. Urine cytology should be performed at each visit and annual cystoscopy should be performed for the first 2-3 years.

In conclusion, primary MALT lymphoma of the bladder represents a rare malignancy with excellent prognosis if detected at an early stage. For early stage disease, definitive radiation represents an excellent treatment modality with a minimal side-effect profile.

## Figures and Tables

**Table 1:** Characteristics of reported cases of primary MALT lymphoma of the bladder on PubMed as of June 2015.

Author	Age	Sex	Presenting symptom	Treatment	Follow-up (Months)	Outcome
Hughes et al., [22]	81	Female	Hematuria	Diathermy TURBT	12	NED
Ando et al., [16]	77	Female	Urinary retention	TURBT	36	NED
Kempton et al., [23]	64	Female	*∗*	Partial cystectomy	144	Died of unknown cause
Fernandez Acenero et al., [24]	73	Female	Dysuria, Back pain	Chemotherapy	8	Died of unknown cause
Fernandez Acenero et al., [24]	50	Female	Fever	Chemotherapy	60	NED
Fernandez Acenero et al., [24]	75	Female	Hematuria, dysuria and Breast carcinoma	Chemotherapy	9	NED
Gallardo et al., [25]	70	Female	Hematuria, Dysuria, Malaise and weight loss	Chemotherapy	24	NED
Morita et al., [26]	68	Female	persistent interstitial cystitis	Chemotherapy [Rituximab]	*∗*	NED
Wazait et al., [27]	65	Female	Hematuria, dysuria and recurrent UTI's	Chemotherapy [CHOP]	36	NED
Hughes et al., [22]	77	Male	Hematuria	Chemotherapy [ChID]	48	NED
Hughes et al., [22]	28	Male	Hematuria	Chemotherapy [ChIVP]	120	NED
Kakuta et al., [17]	84	Female	General fatigue and weight loss	Chemotherapy [R-CHOP]	*∗*	*∗*
Sen et al., [28]	31	Female	Incidental finding - Pregnancy	Postpartum Chemotherapy	*∗*	*∗*
Kempton et al., [23]	79	Female	*∗*	Radiation	24	Died of Myocardial infarction
Al-Maghrabi et al., [10]	64	Female	Hematuria, and recurrent UTI	Radiation	156	NED
Al-Maghrabi et al., [10]	69	Female	Treatment resistant UTI	Radiation	60	NED
Al-Maghrabi et al., [10]	72	Female	Hematuria and recurrent UTI	Radiation	36	NED
Al-Maghrabi et al., [10]	62	Male	Hematuria	Radiation	24	NED
Tsang et al., [29]	*∗*	*∗*	*∗*	Radiation	*∗*	NED
Tsang et al., [29]	*∗*	*∗*	*∗*	Radiation	*∗*	NED
Tsang et al., [29]	*∗*	*∗*	*∗*	Radiation	*∗*	NED
Hughes et al., [22]	76	Female	Hematuria	Radiation	24	NED
Hughes et al., [22]	66	Female	Recurrent UTI	Radiation	*∗*	Deceased
Takahara et al., [30]	85	Female	Hematuria	Radiation	*∗*	NED
Hatano et al., [15]	84	Female	Hematuria and recurrent UTI	Radiation	12	NED
Kempton et al., [23]	73	Female	*∗*	Fulguration and Radiation	120	Died of unknown cause
Kempton et al., [23]	27	Female	*∗*	Tumor resection and Radiation	480	Died of fibrosarcoma
Kempton et al., [23]	45	Male	*∗*	Segmental resection and Radiation	312	Died of unknown cause
Kempton et al., [23]	50	Female	*∗*	Segmental resection and Radiation	240	NED
Ueno et al., [31]	64	Female	Incidental finding	TURBT and Radiation	19	Recurrence Stomach
Haddad-lacle et al., [32]	54	Male	Low back pain, incidental bladder mass	TURBT and Radiation	36	NED
Wazait et al., [27]	70	Female	Hematuria	TURBT-1 yr recurrence, Chemotherapy on recurrence	60	NED
Szopiński et al., [33]	17	Female	Incidental finding	TURBT and Chemotherapy	24	NED
Maninderpal et al., [34]	65	Female	Chronic suprapubic mass, nausea and feeling unwell	TURBT and Chemotherapy	3	Died of Sepsis
Matsuda et al., [3]	78	Female	Refractory Cystitis and renal dysfunction	TURBT and Chemotherapy [rituximab]	*∗*	*∗*
Kawakami et al., [35]	27	Male	*∗*	Radiation and Chemotherapy [doxorubicin]	18	NED
Tasu et al., [36]	75	Female	Hematuria	Radiation and Chemotherapy [cyclophosphamide]	36	NED
Painemal et al., [37]	70	Female	Hematuria, Dysuria, Malaise and weight loss	Radiation and Chemotherapy	48	NED
Hughes et al., [22]	82	Female	Hematuria	Radiation + Chemotherapy [ChIVP]	*∗*	Deceased
Terasaki et al., [19]	64	Female	General malaise and anemia	Radiation and Chemotherapy [Rituximab]	14	NED
Mizuno et al., [38]	72	Female	Hematuria and recurrent cystitis	TURBT, Radiation and Chemotherapy [rituximab]	*∗*	*∗*
Bacalja et al., [39]	48	Male	Incidental finding	TURBT, Radiation and Chemotherapy [R-CHOP]	5	Remission
Mayer et al., [40]	70	Male	Bladder mass with scalp metastasis	Chemotherapy and Radiation for scalp and brain	14	Died of Brain Metastasis
van den Bosch et al., [41]	59	Male	Hematuria	triple therapy [patient denied radiation]	36	NED
Fujimura et al., [42]	69	Female	Hematuria and anemia	Antibiotics and *H. pylori* therapy	24	NED
Terada, [43]	88	Female	Hematuria	Antibiotics	6	Markedly reduced tumor size
Lucioni et al., [20]	72	Female	Persistent dysuria	Antibiotics	6	NED
Bates et al., [11]	66	Female	Bladder mass	*∗*	12	NED
Bates et al., [11]	79	Female	Hematuria	*∗*	*∗*	*∗*
Bates et al., [11]	59	Female	Untreated solid necrotic tumor	*∗*	36	NED
Kröber et al., [44]	57	Male	Obstructive dysuria	*∗*	*∗*	*∗*
Takahashi et al., [30]	71	Female	Hematuria	*∗*	*∗*	*∗*

^*∗*^Authors do not have access to this information.

CHOP: cyclophosphamide, hydroxydaunorubicin, vincristine, and prednisone.

R-CHOP: cyclophosphamide, hydroxydaunorubicin, vincristine, prednisone, and rituximab.

ChlVP: chlorambucil, vincristine, and prednisolone.

TURBT: transurethral resection of bladder tumors.

NED: no evidence of disease.
